# Dual Role of *Alchemilla vulgaris* L. Extract in Breast Cancer Regression: Reestablishment of Effective Immune Response

**DOI:** 10.3390/ph17030286

**Published:** 2024-02-23

**Authors:** Sanja Jelača, Ivan Jovanovic, Dijana Bovan, Marina Z. Jovanovic, Milena M. Jurisevic, Duško Dunđerović, Zora Dajic-Stevanovic, Nebojsa Arsenijevic, Sanja Mijatović, Danijela Maksimović-Ivanić

**Affiliations:** 1 Department of Immunology, Institute for Biological Research “Siniša Stanković”—National Institute of the Republic of Serbia, University of Belgrade, Bulevar Despota Stefana 142, 11108 Belgrade, Serbia; sanja.jelaca@ibiss.bg.ac.rs (S.J.); dijana.draca@ibiss.bg.ac.rs (D.B.); 2 Center for Molecular Medicine and Stem Cell Research, Faculty of Medical Sciences, University of Kragujevac, Svetozara Markovića 69, 34000 Kragujevac, Serbia; ivanjovanovic77@gmail.com (I.J.); nebojsa_arsenijevic@yahoo.com (N.A.); 3 Department of Otorhinolaryngology, Faculty of Medical Sciences, University of Kragujevac, 34000 Kragujevac, Serbia; marina_jovanovic@rocketmail.com; 4 Department of Pharmacy, Faculty of Medical Sciences, University of Kragujevac, 34000 Kragujevac, Serbia; milena.jurisevic13@gmail.com; 5 Institute of Pathology, School of Medicine, University of Belgrade, Dr Subotića 8, 11000 Belgrade, Serbia; dusko.dundjerovic@med.bg.ac.rs; 6 Faculty of Agriculture, University of Belgrade, Nemanjina 6, 11080 Belgrade, Serbia; dajic@agrif.bg.ac.rs

**Keywords:** *Alchemilla vulgaris* L., breast cancer, apoptosis, tumor microenvironment, immune response

## Abstract

Ethnomedicinal records have long mentioned the historical usage of *Alchemilla vulgaris* L. in folk medicine, particularly for the treatment of gynecological issues. Building on this ethnomedicinal knowledge regarding female illnesses, the aim of this research was to evaluate the impact of ethanolic extract of *A. vulgaris* on mouse breast cancer cells (4T1) in vitro and in vivo, in addition to its effect on the immune compartment in the tumor microenvironment. Behind viability decrease of 4T1 cells induced by treatment with *A. vulgaris* extract was strong inhibition of cell proliferation accompanied by caspase-dependent apoptosis and autophagic cell death. Observed changes in 4T1 cell culture after treatment were well orchestrated and led to a reduction in metastatic potential through weakened adhesion, invasion, migration, and colony-forming abilities in vitro. Enhanced intracellular production of reactive oxygen and nitrogen species promoted by the treatment might interfere with all the observed effects. Apart from the direct effect on tumor cells, the *A. vulgaris* extract significantly reduced tumor growth in the solid orthotropic mammary carcinoma model through restitution of efficient local and systemic immune response reflected in enhanced antigen-presenting potential of dendritic cells (DCs) as well as the extent and activity of effector T cells.

## 1. Introduction

Breast cancer stands as the most prevalent form of malignancy among women worldwide and accounts for 24.5% of all malignancies in the female population. Despite better diagnostic skills, it causes around 6.9% of cancer-related deaths. Until recently, lung cancer was considered the most commonly diagnosed type of cancer; however, in 2020, around 2.26 million new cases of breast cancer were recorded, surpassing the number of new lung cancer cases and accounting for 11.7% of all reported cancer cases [1]. As it is a multifactorial disease, various factors such as genetic factors, population structure, environment, and lifestyle contribute to its occurrence [2,3]. The worldwide prevalence of breast cancer has risen due to changes in risk factors [4].

If it is diagnosed at an early stage, the main goal of breast cancer treatment is to cure and prevent the risk of recurrence; thus, therapy involves a radical approach [5]. In the metastatic form, on the other hand, the aim is to achieve control of the disease and as long as possible survival of the patient while maintaining quality of life [6]. Several local and systemic methods are used for treatment. The most commonly used methods are surgical removal, radiation, chemo-, and targeted therapy. Targeted therapy encompasses endocrine treatment for breast cancer with positive hormone status and anti-HER2 therapy for HER2+ breast cancer [7]. Innovative approaches showing promising results include immunotherapy, conjugated antibodies, and targeting metabolic pathways [8].

Predominant histological forms of breast cancer include apocrine, medullary, cribriform, metaplastic, classic lobular, neuroendocrine, mucinous, pleomorphic lobular, and tubular carcinomas. In addition to these types, the most frequently diagnosed cases involve the nonspecific type of invasive ductal carcinoma. Presently, four molecular subgroups, luminal A, luminal B, HER2+, and triple-negative, are widely recognized and firmly established in clinical practice [8,9]. The term triple-negative breast cancer (TNBC) describes a subtype of breast cancer in which expression of the three primary breast tumor markers, including estrogen receptor (ER), progesterone receptor (PR), and HER2 protein, is absent. This form of breast cancer constitutes approximately 15 to 20% of all breast cancer cases and approximately 40% of high-grade tumors [10]. TNBC is of particular interest to researchers as it poses a therapeutic challenge mainly due to its poor response to therapy and highly invasive nature [11].

Apart from the initial diversity of neoplastic transformation, cancer also includes many other cell types in addition to tumor cells, such as adipocytes, fibroblasts, endothelial cells, and immune cells (macrophages and lymphocytes), as well as the extracellular matrix’s non-cellular constituents (fibronectin, collagen, hyaluronan, laminin, etc.). All of them create the network of the tumor microenvironment (TME) [12]. Tumor cells and other components of the TME are in constant, reciprocal communication. Normally, the immune system acts as a defense mechanism against foreign entities, including tumors, aiming to prevent their growth and metastasis. However, many reports have shown that tumor-infiltrating immune cells can also be involved in tumor progression in many ways. Apart from well-known tumor immune evasion strategies, the TME architecture and extracellular matrix construction enables recruitment of tumor suppressors such as MDSCs and Tregs that orchestrate the establishment of local immunosuppression [13,14,15]. Considering the tumor tissue as a never-healing wound, control points of immune response regulation support the entry of effector cells into the exhaustion state [16]. Therefore, cancer therapy should not only aim to destroy tumor cells but also trigger the flow of events leading to refreshed antitumor immune response.

Despite many treatment options, recurrence and accompanying problems persist among patients, primarily due to many side effects, general toxicity, and aggressive behavior of tumors. Lately, the use of some types of phytotherapy in breast cancer patients has increased significantly [17]. Medicinal plants are becoming more and more popular as they are recognized as efficient and affordable sources of new chemotherapeutic agents [18]. Following the rule that nature is best complemented and balanced by nature itself, the potential of naturally occurring compounds to interact directly or indirectly with host cells opens up numerous opportunities to influence the tumor ecosystem. When it comes to the complex mixture of compounds in the organic extract, two types of interactions and the crosstalk between them may be crucial—a direct effect on tumor cells and the potential to affect the phenotype and function of immune cells. The immunogenic death of tumor cells induced by various plant polyphenols leads to the release of danger signals and the subsequent refreshment of the antitumor immune response through enhanced potential of antigen-presenting dendritic cells and effector T-cell responses [16]. Finally, natural products can disrupt the inhibitory signals responsible for creating an immunosuppressive environment in tumors and refresh T-cell effector performance.

Among many plants that exert a variety of biological activities is *Alchemilla vulgaris*, widely known as lady’s mantle, as many ethnomedicinal reports mention its well-known effects against disorders of the female reproductive system (pruritus vulvae, dysmenorrhea, heavy menstrual flow, menopausal complaints). In addition, its activity against reproductive system-related diseases (endometriosis, cysts, fibroids, and infertility) has been described [19,20,21,22,23]. The anticancer activity of this plant has been reported against tumors of the female reproductive organs (A2780 and HeLa), as well as against human prostate cancer (PC-3), human breast cancer (MCF-7), human melanoma A375, human lung carcinoma A549, and human colorectal cancer (Caco2, HCT116) cell lines in vitro [24,25,26]. Our previous research showed strong anticancer properties of ethanolic extract of *A. vulgaris* against non-invasive and highly invasive murine melanoma [27]. Based on ethnomedicinal data on female disorders and illnesses, the objective of this research was to evaluate the direct activity of *A. vulgaris* extract against murine triple negative breast cancer cells (4T1) in an in vitro and in vivo setting, along with its effect on the tumor microenvironment in an ex vivo setting. The data obtained showed that in addition to the direct antitumor effect of the *A. vulgaris* extract realized through inhibition of cell division, apoptotic cell death and diminished metastatic features, tested extract exerted a strong impact on the systemic and local immune response. Considering all of the above points, we conclude that *A. vulgaris* extract reduces tumor growth through a direct effect on the viability of tumor cells and reestablishment of an effective antitumor immune response.

## 2. Results

### 2.1. A. vulgaris Ethanolic Extract Exhibits Strong Antitumor Properties against All Cell Lines In Vitro

In order to evaluate the antitumor potential of *A. vulgaris* ethanolic extract against the murine mammary carcinoma (4T1) and human breast adenocarcinoma (MDA-MB-468, MDA-MB-231, and MDA-MB-361) cell lines, cells were treated with a wide range of concentrations of ethanolic extract of *A. vulgaris*. The determination of viability was conducted using MTT and SRB assays after 72 h incubation (Table 1). As presented in Figure S1, the *A. vulgaris* extract dose-dependently decreased viability of all cell lines used in this study. In concordance with previous data, the viability of primary peritoneal exudate cells derived from the peritoneal cavity of C57BL/6 mice was fully preserved after treatment with *A. vulgaris* extract [26], indicating a high selectivity of the tested extract for the malignant phenotype. 

### 2.2. A. vulgaris Extract Induces Programmed Cell Death Type I and II in 4T1 Cell Line

**Figure 1:**
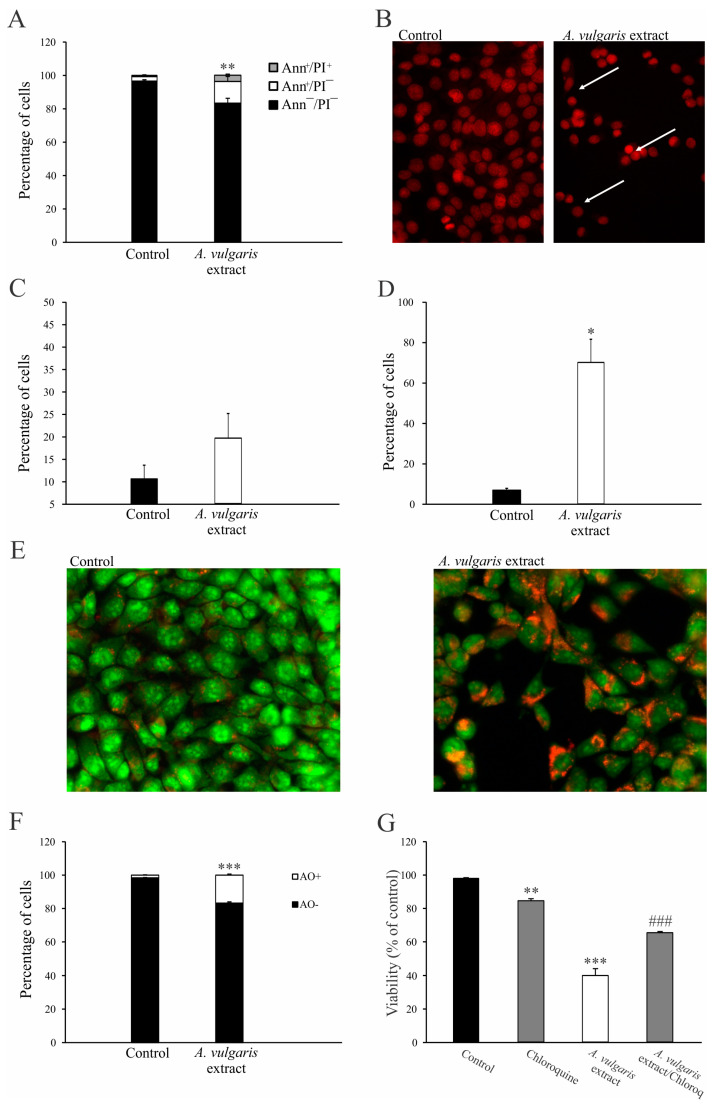
*A. vulgaris* extract induces programmed cell death I and II. The 4T1 cell line was incubated with *A. vulgaris* extract (IC_50_ concentration) for 72 h, and flow cytometry and fluorescence microscopy (400× magnification) were performed. Ann/PI staining (**A**), PI staining on chamber slides (apoptotic nuclei are indicated by white arrows) (**B**), apostat staining (**C**), DHR 123 staining (**D**), acridine orange (AO) staining (**E**,**F**), and viability determination via SRB (**G**) were carried out. Results are presented as mean ± SD of three independent experiments and statistical significance was determined using the Student *t*-test. * *p* < 0.05; ** *p* < 0.01; *** *p* < 0.001 compared with untreated control. ### *p* < 0.001 compared with *A. vulgaris* extract treatment.

To investigate the potential mechanism of action, mouse breast cancer cells (4T1) were treated with an IC_50_ concentration of *A. vulgaris* extract and incubated for 72 h. Thereupon, flow cytometric analysis revealed that the majority of cells detected after treatment with the *A. vulgaris* extract exhibited features of early apoptosis, as shown by the Ann^+^/PI^−^ profile (Figure 1A and Figure S3A). Microscopic examination confirmed this result and showed typical morphological signs of apoptosis after PI staining (Figure 1B). The observed apoptosis was accompanied by only slight caspase activity in the treated cells (Figure 1C, Figures S2 and S4B). Considering that free radicals are known mediators of cellular damage that ultimately lead to cell death when their production overcomes redox protection, the impact of the tested extract on their generation was investigated. Cytofluorimetric and microscopic analysis showed a potent stimulation of intracellular ROS/RNS production, confirming that these molecules are mediators of the observed cytocidal effects of the *A. vulgaris* extract (Figure 1D, Figures S3 and S4C). In the same time frame, *A. vulgaris* extract strongly promoted autophagy, which was visualized with fluorescence microscopy and quantified via flow cytometry (Figure 1E,F and Figure S4D). In addition, concomitant treatment with *A. vulgaris* extract and the specific autophagy inhibitor chloroquine (20 µM) resulted in an increase in cell survival by approximately 30% in comparison to cells treated with *A. vulgaris* extract alone (IC_50_ concentration), confirming that the autophagic process serves as a route for cell elimination (Figure 1G). In summary, *A. vulgaris* extract induced both type I and type II programmed cell death with a strong oxidative burst in the background.

### 2.3. A. vulgaris Extract Abrogates the Metastatic Potential of 4T1 Cell Line

**Figure 2:**
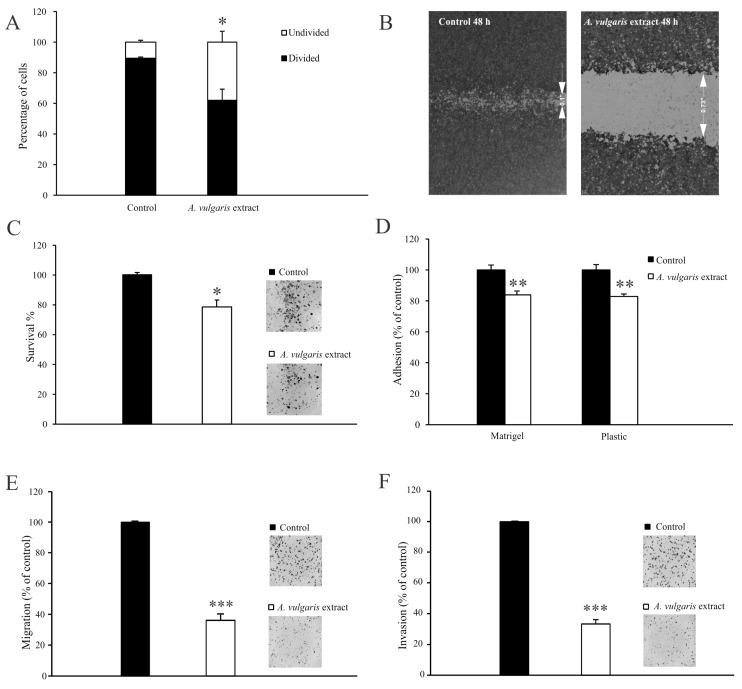
*A. vulgaris* extract abolishes the metastatic potential of the 4T1 cell line. Cells prestained with CFSE and exposed to an IC_50_ concentration (45 μg/mL) of *A. vulgaris* extract were analyzed via flow cytometry analysis after 72 h (**A**). Cells exposed to a subtoxic concentration (22.5 μg/mL) of *A. vulgaris* extract (**B**–**F**). Wound healing assay after 48 h (**B**). Colony formation (**C**), adhesion (**D**), migration (**E**), and invasion assays (**F**) were carried out after 72 h of cultivation. Results (**C**–**F**) are expressed as percentage of untreated cells (control). The data are displayed as mean ± SD calculated from three independent repeats and statistical significance was determined using the Student *t*-test. * *p* < 0.05; ** *p* < 0.01; *** *p* < 0.001 compared with untreated control.

It is widely recognized that 4T1 cells exhibit a high proliferation rate, which is one of the features closely related to their aggressive phenotype. To explore the potential antimetastatic effects of the *A. vulgaris* extract, a series of in vitro assays were conducted. Flow cytometric analysis revealed the loss of dividing potential of 4T1 cells after exposure to *A. vulgaris* extract (Figure 2A and Figure S3E). Evaluation of cell motility using a wound-healing assay revealed that after a 48 h treatment of 4T1 cells with a subtoxic dose (22.5 μg/mL) of *A. vulgaris* extract, the wound area was significantly preserved compared with the control group (Figure 2B). In contrast, the control cells completely filled the gap within the same time frame. The average width of the wound in the control was 0.11 ± 0.015 inches compared with 0.7 ± 0.02 inches after treatment, indicating a strong antimigratory potential of *A. vulgaris* extract (*p* = 0.000004). In parallel, the colony formation potential of the treated cells decreased by about 20% (Figure 2C). Cell adhesion to both plastic surfaces and the extracellular matrix (Matrigel^®^) was strongly affected by *A. vulgaris* treatment (Figure 2D). Furthermore, *A. vulgaris* extract reduced the ability of 4T1 cells to pass through 8 μm pores with or without extracellular matrix layer by approximately 60% compared with the control (Figure 2E,F), confirming that both migratory and invasive potential were affected.

Overall, the loss of dividing potential of 4T1 cells together with significant decreases in colony formation potential, adhesion, migration, and invasion indicates the change in the metastatic features of these cells after *A. vulgaris* extract treatment.

### 2.4. A. vulgaris Extract Downregulates the Protein Expression Profile in the Metastatic Process

**Figure 3:**
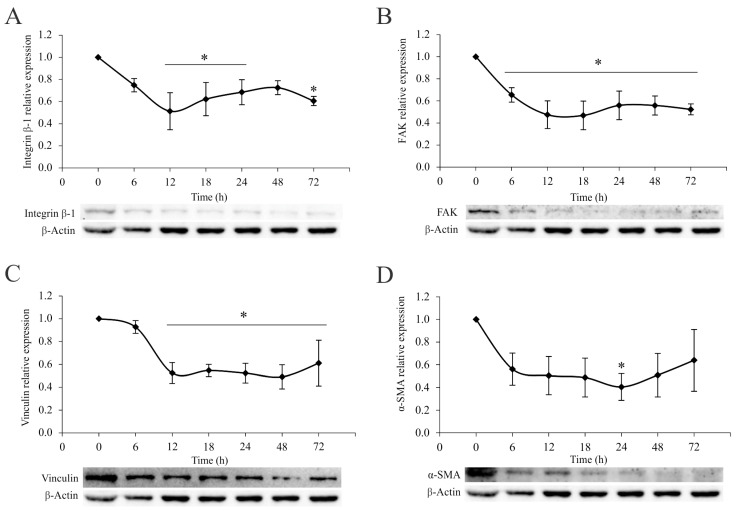
*A. vulgaris* extract downregulates the integrin signaling pathway. Relative expression of integrin β-1 (**A**), FAK (**B**), vinculin (**C**), and α-SMA (**D**) in 4T1 cells after the treatment with *A. vulgaris* extract at the indicated time points, analyzed by Western blot method. The results are expressed as mean ± SD based on three independent experiments, and * *p* values of less than 0.05 were considered statistically significant compared with the control. Statistical significance was assessed by one-way ANOVA followed by Tukey’s honest significant difference (HSD) post hoc test.

To confirm the diminished metastatic potential of 4T1 cells at the intracellular level, the impact of the *A. vulgaris* extract on the signaling pathways relevant for the metastatic cascade was examined. Data in the literature confirm that the invasiveness and migratory potential of tumor cells depend on the engagement of integrin receptors and subsequent downstream signaling cascades. Thus, 4T1 cells were treated with an IC_50_ concentration of *A. vulgaris* extract for 6, 12, 18, 24, 48, and 72 h and protein expression was analyzed via the Western blot method. The results showed a continuous decrease in the relative expression of integrin β-1, focal-adhesion kinase (FAK), and vinculin, while α-smooth muscle actin (α-SMA), a protein associated with an aggressive phenotype, showed a similar trend but with less significance compared with untreated cells (Figure 3D). As mentioned above, the intracellular protein signature confirmed the abolishment of metastatic potential by the *A. vulgaris* extract.

### 2.5. A. vulgaris Extract Suppresses Tumor Growth In Vivo

**Figure 4:**
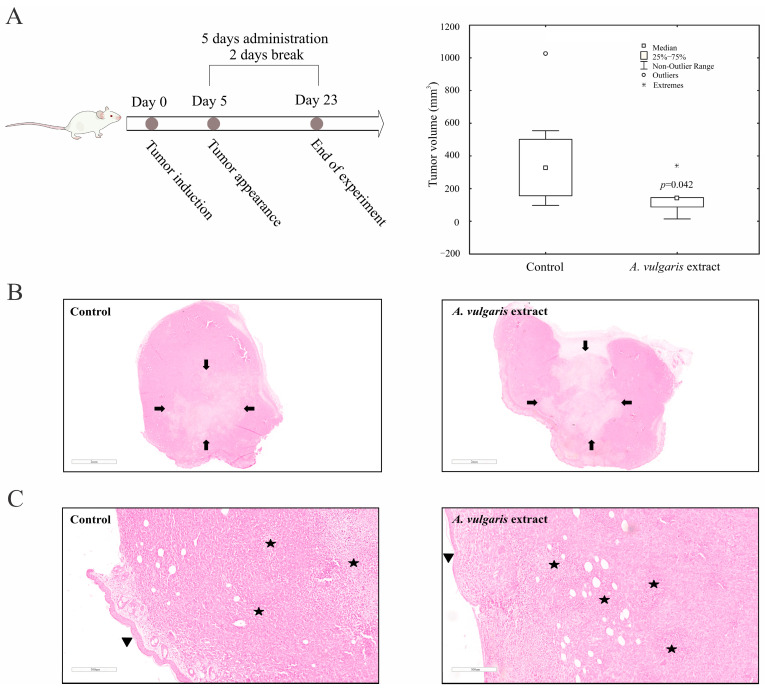
*A. vulgaris* extract decreases tumor volume in vivo. 4T1 cells were inoculated into BALB/C mice and treated with 50 mg/kg *A. vulgaris* extract. Tumor volume was determined after 23 days. * *p* < 0.05 was considered statistically significant, compared with control (**A**). Control tumor tissue, and tumors derived from *A. vulgaris* extract-treated group (**B**), and (**C**). ((**B**): 12× magnification; (**C**): 100× magnification; H/E staining.) Arrows indicate the macroscopic areas of necrosis, while stars denote microscopic areas of apoptonecrosis. Arrowhead points toward epidermis which lies on the surface above the tumor tissue.

To define the effect of the *A. vulgaris* extract in vivo, tumors were induced in BALB/C mice by orthotopic implantation of 4T1 cells and treatment was started as soon as the tumors became palpable. The *A. vulgaris* extract significantly decreased tumor volume compared with the control group (*p* = 0.042) (Figure 4A). Histopathological analysis of the tumor tissue showed that the amount of necrosis was more extensive in the group treated with *A. vulgaris* extract compared with the control (30% and 25%, respectively). Necrosis occurred mostly in the form of geographic areas, and to a lesser extent in the form of necroptotic foci (around 2% in the control group and less than 1% in the treated group). Despite the significantly reduced tumor volume, no visual changes in the shapes and sizes of tumor cells among the groups were observed under the light microscope (Figure 4B,C).

**Figure 5:**
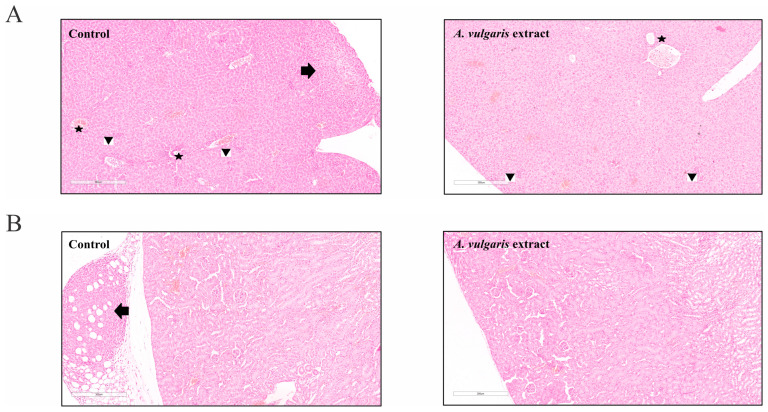
*A. vulgaris* extract treatment showed no signs of tissue toxicity. (**A**) Liver tissue of the control group and *A. vulgaris* extract-treated group (100× magnification; H/E staining) with arrow pointing to metastasis, stars marking portal tracts, and arrowheads showing sinusoidal extramedullary hematopoiesis, and (**B**) kidney tissue of the control group and *A. vulgaris* extract-treated group (100× magnification; H/E staining). Arrow denotes metastasis to the perirenal adipose tissue. No significant toxic changes were observed in either group.

The samples of liver tissue in both groups showed mostly the same morphological changes. Sinusoidal extramedullary hematopoiesis with focal dilatation of venules was registered, while the presence of inflammation or hepatocyte degeneration was not observed. Importantly, the presence of subcapsular metastatic deposits was detected in the control group but not in the tissue samples of the *A. vulgaris* extract-treated group (Figure 5A). In addition, no signs of tissue toxicity as such atrophy, inflammation, edema, or cell death signatures were seen in either group. Similar to the liver sections, the presence of metastatic foci was detected in the para-capsular region in the few control kidney tissue samples (Figure 5B). Parameters of toxicity were not identified in the urine of the animals after treatment with *A. vulgaris* extract (Table S1), which is in accordance with the preservation of kidney morphology. Summarizing all the data obtained, it can be concluded that *A. vulgaris* exerts a strong tumoricidal potential with minimal systemic toxicity.

### 2.6. Administration of A. vulgaris Extract Increases the Percentage of and Activates Dendritic Cells in the Spleen and Tumor Microenvironment

**Figure 6:**
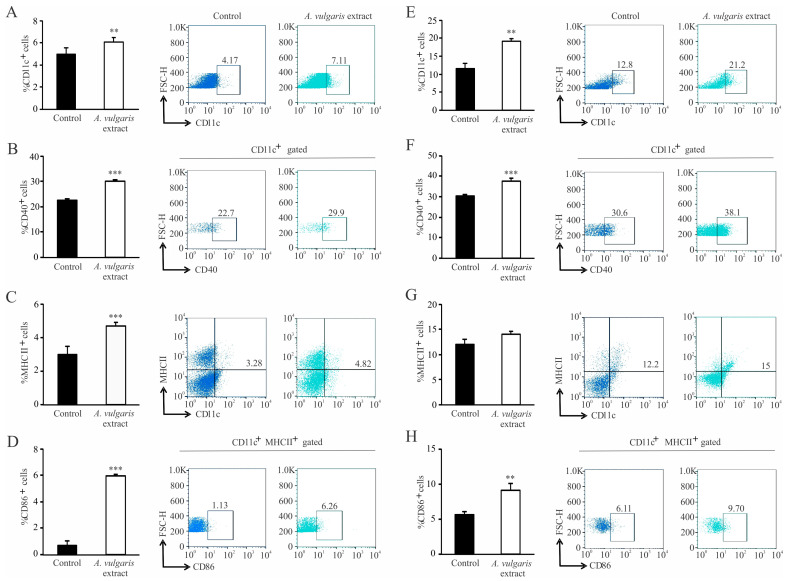
Dendritic cells’ phenotype change in the spleen and in the primary tumor of treated mice. Left panel: The graphs alongside representative FACS plots displaying CD11c^+^ cells’ percentage (**A**) and the CD11c^+^CD40^+^ percentage (**B**), CD11c^+^MHCII^+^ (**C**) and CD11c^+^MHCII^+^CD86^+^ cells (**D**) in the spleen. Right panel: The graphs with representative FACS plots displaying the CD11c^+^ cells’ percentage (**E**) as well as the CD11c^+^CD40^+^ percentage (**F**), CD11c^+^MHCII^+^ (**G**), and CD11c^+^MHCII^+^CD86^+^ cells (**H**) in the primary tumor tissue. Data are presented as mean ± SEM of six mice per group and are illustrative of three separate experiments. Statistical significance was assessed by Mann–Whitney rank sum test or Student’s unpaired *t*-test, where applicable (** *p* < 0.01; *** *p* < 0.001).

Further, the impact of the *A. vulgaris* extract treatment on the tumor microenvironment was assessed. After tumor induction, administration of *A. vulgaris* extract significantly increased the percentage of CD11c^+^ dendritic cells in the spleen and primary tumor, as shown in Figure 6A,E. Alongside this, administration of *A. vulgaris* extract significantly increased the percentages of CD40^+^ (Figure 6B) and MHCII^+^ (Figure 6C) as well as MHCII^+^CD86^+^ dendritic cells in the spleens of tumor-bearing mice (Figure 6D). When it comes to the tumor microenvironment, *A. vulgaris* extract significantly increased the percentage of CD40^+^ dendritic cells (Figure 6F). A similar trend was observed for the percentages of MHCII^+^ (Figure 6G) and MHCII^+^CD86^+^ dendritic cells (Figure 6H).

### 2.7. A. vulgaris Extract Elicits Acquired Immune Response in the Spleen and Within the Primary Tumor by Activating Cytotoxic T Lymphocytes

**Figure 7:**
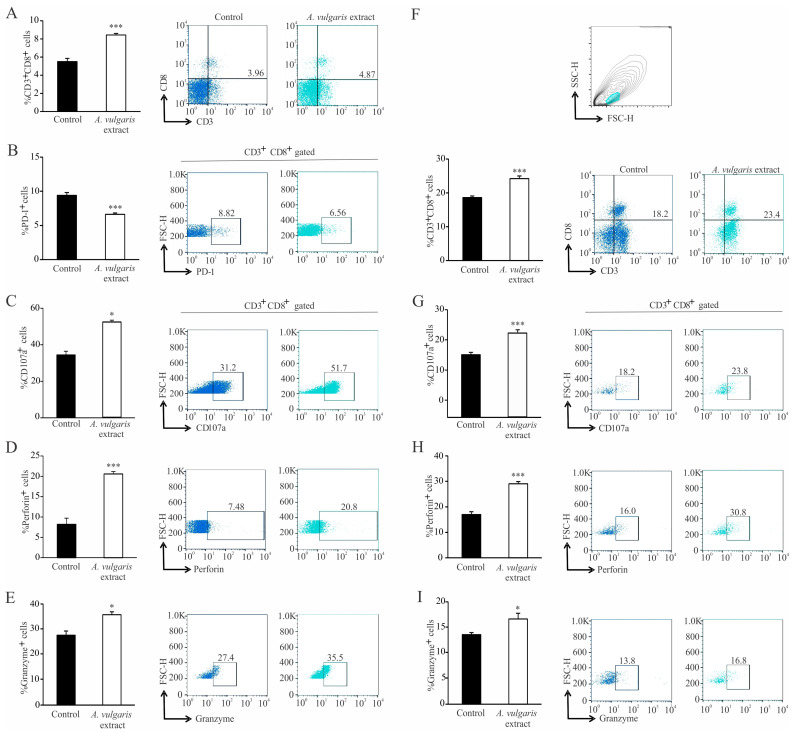
Activation of cytotoxic T lymphocytes in the spleen and primary tumor in treated mice. Left panel: The graphs and illustrative FACS plots displaying the CD3^+^CD8^+^ cells’ percentage (**A**) and also the PD-1^+^ percentage (**B**), CD107a^+^ (**C**), perforin^+^ (**D**), and granzyme^+^ CD3^+^CD8^+^ cells (**E**) in the spleen. Right panel: The graphs and illustrative FACS plots displaying the CD3^+^CD8^+^ cells (**F**), CD107a^+^ (**G**), perforin^+^ (**H**), and granzyme^+^ CD3^+^CD8^+^ cells’ percentage (**I**) in the tumor microenvironment. Data are presented as mean ± SEM of six mice per group and are illustrative of three separate experiments. Statistical significance was assessed by Mann–Whitney rank sum test or Student’s unpaired *t*-test, where applicable (* *p* < 0.05; *** *p* < 0.001).

Administration of *A. vulgaris* extract significantly increased the percentage of CD3^+^CD8^+^ T lymphocytes in the spleens of tumor-bearing mice (Figure 7A). In addition, the percentage of CD3^+^CD8^+^PD-1^+^ was significantly decreased (Figure 7B). On the other hand, the percentage of CD107a^+^, perforin^+^, granzyme^+^, CD3^+^CD8^+^ T lymphocytes was significantly increased in the spleen of tumor-bearing mice in response to *A. vulgaris* extract (Figure 7C–E).

In the tumor microenvironment, the percentage of CD3^+^CD8^+^ was significantly increased in the treated mice (Figure 7F). Moreover, the percentage of CD107a^+^, perforin^+^, and granzyme^+^ CD3^+^CD8^+^ T lymphocytes was also significantly increased in the primary tumor after administration of *A. vulgaris* extract (Figure 7G–I).

## 3. Discussion

Ethnobotanical data refers to the traditional knowledge and practices of various cultures regarding the use of plants and plant-based remedies for medicinal purposes. Throughout history, different societies have relied on inherited knowledge passed down from generation to generation for treatment of various ailments, including cancer. Breast cancer, being one of the foremost prevalent cancer types affecting women, is also the subject of research in the world of ethnobotanical medicine. Ethnobotanical data can provide valuable insights and serve as a starting point for further scientific research and the discovery of novel compounds that may have therapeutic potential and/or improve current protocols, mainly through the sensitization of tumor cells to radio- or chemotherapy, or refreshment of immune cell activities against the malignant phenotype [28,29,30,31]. Importantly, several plants are traditionally used in various cultures to treat breast cancer symptoms and improve overall well-being [18,29,32,33]. Some of them are sources of drugs that are successfully used in modern chemotherapy for breast cancer treatment, such as paclitaxel [34,35]. In general, rigorous scientific research has been necessary to confirm the safety and effectiveness of these plant-derived remedies against breast carcinoma.

The literature data report well-known effects of *Alchemilla vulgaris* L. against female reproductive system disorders and related diseases, as well as its use in the treatment of diabetes, multiple sclerosis, anemia, skin rashes, hernias, ulcers, wounds, and inflammations [19,20,21,22,36]. Our previous results showed the anticancer potential of *A. vulgaris* extract against a panel of human cancer cell lines in vitro as well as against murine melanoma of different aggressiveness in vivo [26,27]. 

In the current research, the impact of *A. vulgaris* ethanolic extract was investigated on a panel of breast cancer cell lines, starting from the human ER+ breast cancer cell line (MDA-MB-361) and continuing with TNBC cell lines of human (MDA-MB-468 and MDA-MB-231) and murine origin (4T1). In concordance with previously reported data, *A. vulgaris* extract treatment dose-dependently decreased the cell viability of all tested cells observed with both MTT and SRB assays [26,27]. Selectivity toward the malignant phenotype has already been described with no effect against primary cells extracted from the peritoneal cavity of C57BL/6 mice [26]. Concordantly, Ibrahim et al. reported that methanolic extract of *A. vulgaris* root showed no influence on normal Vero cells [25]. To allow transfer of the in vitro study to in vivo, the drug efficacy was further tested in detail on the highly aggressive breast cancer cell line 4T1, enabling the subsequent assessment of the extract’s antitumor potential in a syngeneic animal model. This approach covers multiple aspects of the extract’s influences on solid tumor formation and tumor microenvironmental features [37]. To reach this goal, it was essential to define the main routes of direct influence of the extract in cell culture. Flow cytometric analysis and fluorescence microscopy showed that *A. vulgaris* extract induced apoptosis of 4T1 cells accompanied by moderate caspase activation, which was in line with previous data showing a similar mode of action on human lung cancer cells (A549) and highly invasive murine melanoma cells (B16F10) [26,27]. The literature data report many natural compounds isolated from plants with apoptosis-inducing potential [38,39,40]. The significant autophagy observed in 4T1 cells exposed to *A. vulgaris* extract was abolished with concomitant treatment with the autophagy inhibitor chloroquine, resulting in restored cell viability, verifying once more the contribution of this process to the tumor-cell-destructive activity of *A. vulgaris* extract [26,27]. The strong oxidative burst provoked by the treatment may be responsible for the tumoricidal activities of the *A. vulgaris* extract compounds’ mixture and their interplay.

The 4T1 cells are a well-known model considered to be one of the most formidable forms of breast cancer with strong potential to form distant metastases in various organs [41,42]. In line with our previous data, *A. vulgaris* extract treatment inhibited proliferation of 4T1 cells [26,27], and significantly reduced their ability to fill a gap or migrate through porous membranes when attracted by chemo-stimuli. In addition, the potential of 4T1 cells to pass the reconstructed basal membrane barrier was also considerably diminished, confirming that the treatment affected the invading capacity of cells, predicting decreased penetration of cells upon exposure to *A. vulgaris* extract in surrounding tissues in vivo. Altogether, pretreatment with a subtoxic dose of *A. vulgaris* extract, which did not affect the viability of 4T1 cells, markedly affected all features important for the metastatic process—adhesiveness, migration, invasion, and colony forming potential. Simultaneously, the relative expression of integrin β-1, FAK, vinculin, and α-SMA proteins was inhibited by the treatment. According to the literature, adhesion to the ECM is the key event in the initiation of the metastatic cascade [43]. The proteins that connect the ECM to the intracellular cytoskeleton play an important role in adhesion, migration, and invasion. The literature supports the fact that the migratory potential of cells and the invasiveness of tumors depend on the ligation of integrin receptors and the transmission of a downstream signal that links the extracellular compartment to the intracellular cytoskeleton, thus enabling adequate response to dictate from the outside [44,45]. Proteins involved in this intimate contact are part of complex structures called focal adhesions, whose formation and separation are dynamically regulated during the process of cell migration. Inside the cell, the cytoskeleton represents a protein network that defines its morphology and behavior [43,46]. Overexpression of integrin β-1 is a poor prognostic factor for cancer patients [47]. In contrast, negative regulation of integrin β-1 is a good sign and a desirable therapeutic response, as observed upon the exposure to *A. vulgaris* extract, affecting every further step of the metastatic process. Focal adhesion kinase is the key regulator of survival, proliferation, migration, and invasion. This kinase transmits signals from focal adhesions to the cellular cytoskeletal machinery [46]. It is well known that FAK overexpression is linked to an extensive range of human epithelial cancers. Furthermore, the level of FAK expression correlates with tumor invasive potential and poor prognosis [48,49]. Accordingly, in this study, continuous inhibition of FAK relative expression after *A. vulgaris* treatment was detected. Vinculin is an important part of focal adhesion, and perturbations of vinculin–actin interaction impact cell motility, morphology, and adhesion [50]. Its suppression upon exposure to the tested extract is tightly related to the diminished migratory and invasive properties of 4T1 cells. Finally, *A. vulgaris* inhibits the expression of α-SMA, the protein associated with the aggressive phenotype and poor survival of patients with advanced forms of cancer, thus silencing the aggressive nature of 4T1 cells [51,52,53]. 

The fact that a tumor is not a simple group of highly proliferating cells with an abnormal phenotype, but a multicellular structure which functions at an impressively organized level [12,54,55], leads to the conclusion that any in vitro data are of limited value until they are confirmed by adequate in vivo screening. The greatest advantage of the syngeneic model relates to preserved constituents and activities in the TME, and the whole-body support in combating disease, including systemic immunity and non-immune cell activities relevant to the therapeutic response [37]. Thus, the antitumor action of *A. vulgaris* extract was investigated in an orthotopic syngeneic mammary carcinoma model, as the most appropriate for simulating stage IV breast cancer behavior. The applied regime of the *A. vulgaris* extract treatment led to a significant decrease in tumor growth. Similarly, the same effect was noted in non-invasive and highly invasive melanoma models in vivo [27]. The TME, including the innate and acquired immune response, significantly contributed to the efficacy of the applied antitumor therapy [56]. The assessment of the *A. vulgaris* extract’s influence on systemic and local immune cell activities in the TME of the 4T1 breast cancer model revealed multiple and well-orchestrated changes, affecting tumor progression and spread. Dendritic cells are professional antigen-presenting cells that trigger and shape the immune response [57,58]. Upon recognition of antigen, they interact with effector cells, such as NK, NKT cells, and T lymphocytes, and tend to navigate the latter course of overall immune response [59,60]. The importance of dendritic cells is also well illustrated in antitumor immune response [61], as it is known that dendritic cells are the first cells to encounter tumor antigens, migrate towards lymph nodes, and provide activating signals for effector cells (“cancer-immunity cycle”) [62]. Of course, various malignancies have developed defense mechanisms to escape recognition by dendritic cells, either by expressing inhibitory signals or by metabolic impairment [63,64]. The application of *A. vulgaris* ethanolic extract significantly increases the percentage of dendritic cells and potently stimulates their maturation, which is illustrated by a remarkable elevation of CD40, CD86, and MHCII molecules. Identical changes in dendritic cell phenotype were observed in the spleen and in the tumor microenvironment. These results might imply that *A. vulgaris* extract could be equally important for both the initiation and maintenance of antitumor immune response to breast carcinoma. As is already known, 4T1 breast cancer cells are considered to be weakly to moderately immunogenic, and as such, activation of cytotoxic lymphocytes might be hindered [65]. Lately, the low number of dendritic cells and their immature forms in tumor tissue has been thought to be another hallmark of a weakly immunogenic tumor [57]. The results presented in this study show that administration of *A. vulgaris* extract might raise the immunogenicity of a tumor, making the antitumor immune response more efficient. There is growing evidence that naturally occurring compounds are potent inducers of immunogenic cell death, triggering the release of danger-associated molecules, and therefore serve as immunoadjuvants [16]. This cascade of events amplifies the two branches of the immune response, innate and adaptive. The strong immunogenic outcome of treatment with *A. vulgaris* extract can be related to the complexity of its content and the pleotropic effect of its bioactive ingredients. When it comes to cytotoxic T lymphocytes, the administration of *A. vulgaris* extract increases the percentage of CD3^+^CD8^+^ cells in the spleen and tumor tissue. In addition to increased frequency of cytotoxic T lymphocytes in the spleen and tumor microenvironment, the phenotype of these cells is also altered to a more cytotoxic one, as illustrated by significant increment of the degranulation marker CD107a followed by significant increase in perforin and granzyme production. In addition, the expression of the inhibitory marker, PD-1, is significantly lower in splenic cytotoxic T lymphocytes, which implies a positive effect of the *A. vulgaris* extract on the activation of splenic cytotoxic T lymphocytes. Histopathological analysis showed enhanced tumor necrosis in the primary tumor of *A. vulgaris*-treated mice. The existence of tumor necrosis may straighten an antitumor immune response [66], as necrosis promotes the maturation of dendritic cells [67,68]. *A. vulgaris* extract may stimulate antitumor immunity in at least two ways, by directly activating dendritic and cytotoxic T cells, and indirectly via tumor necrosis facilitation of dendritic cells’ maturation, then further activating cytotoxic T lymphocytes.

## 4. Materials and Methods

### 4.1. Reagents and Cells

The reagents and the cells were supplied by the manufacturers listed below: Capricorn Scientific GmbH (Hessen, Germany)—fetal bovine serum (FBS) and culture medium RPMI-1640; Sigma (St. Louis, MO, USA)—ethylenediamine tetraacetic acid (EDTA), phosphate-buffered saline (PBS), Triton X-100, dimethyl sulfoxide (DMSO), Fluoromount-G, Trypsin, RNase, carboxyfluoresceindiacetate succinimidyl ester (CFSE), propidium iodide (PI), trisaminomethane hydrochloride (TRIS HCl), dithiothreitol (DTT), glycerol, phenylmethylsulfonyl fluoride (PMSF), sulforhodamine B (SRB), chloroquine, and acridine orange (AO); Biowest (Riverside, MO, USA)—crystal violet (CV); BD (Pharmingen, San Diego, CA, USA, SAD)—annexin V-FITC (AnnV); BD Bioscience (Bedford, MA, USA)—Matrigel^®^; Thermo Fisher Scientific (Waltham, MA, USA)—dihydrorhodamine 123 (DHR123); Fresenius Kabi AG Germany (Bad Homburg, Germany)—paclitaxel (PCT); Biological Industries (Cromwell, CT, USA)—the penicillin–streptomycin solution; Serva (Heidelberg, Germany)—paraformaldehyde (PFA) and N,N,NN-tetramethylethylenedi (TEMED); AppliChem (St. Louis, MO, USA)—bovine serum albumin (BSA) and 3-(4,5 dimethythiazol-2-yl)-2,5-diphenyltetrazolium bromide (MTT); AppliChem (Darmstadt, Germany)—Tween 20 and sodium dodecyl sulfate (SDS); R&D Systems (Minneapolis, MN, USA)—ApoStat; Lach-Ner (Neratovice, Czechia)—sodium hydroxide (NaOH). American Type Culture Collection (ATCC, Manassas, VA, USA)—human breast adenocarcinoma (MDA-MB-231, MDA-MB-468, and MDA-MB-361).

Mouse breast cancer (4T1) cells were provided as a gift from Prof. Dr. Ludger Wessjohan from the Leibniz Institute of Plant Biochemistry, Halle (Saale), Germany. This cell line was primarily purchased from ATCC. 

All cell lines used in this study were routinely maintained in HEPES-buffered RPMI-1640 medium. The medium was supplemented with 2 mM L-glutamine, 0.01% sodium pyruvate, penicillin, and streptomycin (100 units/mL and 100 μg/mL, respectively) and 10% heat-inactivated FBS. Cells were stored in the incubator (37 °C, 5% CO_2_). For viability determination in 96-well plates, cells were seeded in the following densities: 4T1, MDA-MB-361, and MDA-MB-468 cells (4 × 10^3^ cells/well), and MDA-MB-231 cells (7 × 10^3^ cells/well). For flow cytometry, 4T1 cells were seeded in 6-well plates at a density of 1 × 10^5^ cells/well.

The detailed chemical composition of the ethanolic extract of *Alchemilla vulgaris* L. used in this study and the extraction process was as previously reported [26]. The plant material was collected from the species’ natural habitat—the moderately humid hilly-mountainous grassland in the south-eastern part of Serbia. The above-mentioned plant parts were collected in the flowering phase. Typical samples were taxonomically identified and stored as voucher specimens (No. RS-120718-1). The plant material was air-dried and extracted for two hours in hot ethanol. The chemical composition of the solid, vacuum-evaporated extracts was determined by UHPLC-HRMS. A total of 45 compounds were identified, mainly belonging to the flavonol and flavone glycosides with a dominance of quercetin and kaempferol derivatives [26]. The ethanolic stock solution of *A. vulgaris* was dissolved in pure DMSO (100%) at a concentration of 200 mg/mL. The stock solution was prepared fresh before every usage and the final DMSO concentration in the working solutions did not exceed 0.1% (in vitro) or 4% (in vivo) at the highest concentration. 

### 4.2. Animals

The mice used in this study (BALB/C strain, six to eight weeks old, female) were obtained from the Institute for Biological Research “Siniša Stanković”—National Institute of the Republic of Serbia, University of Belgrade. The animals were kept in the animal facility under standard laboratory conditions (free of specific pathogens, ad libitum food and water consumption). The handling of the mice and the protocols used in the in vivo study were aligned with the rules of European Community guidelines (EEC Directive of 1986; 86/609/EEC). Additionally, the local Institutional Animal Care and Use Committee (IACUC) authorized all study protocols. Finally, the experimental protocols were authorized and granted by the national licensing committee at the Department of the Animal Welfare, Veterinary Directorate, of the Ministry of Agriculture, Forestry and Water Management of the Republic of Serbia (permission No. 323-07-12008/2020-05).

### 4.3. Colorimetric Assays for Cellular Viability

Mitochondrial dehydrogenase activity was assessed through the reduction of MTT to formazan. All cells were first seeded overnight and afterward exposed to *A. vulgaris* extract and PCT (as a positive control). After the incubation period (72 h), the cells were washed twice with 200 μL PBS and cultivated in the presence of MTT solution with a final concentration of 0.5 mg/mL and kept in the incubator (37 °C) until the solution turned from yellow to brown. Following this, the dye was removed and DMSO was added to dissolve the formed formazan. Using an automatic reader for microtiter plates (LKB 5060-006, LKB, Vienna, Austria), the absorbance was measured at 540/670 nm. Cell viability was then expressed as a percentage relative to the control (untreated cells) [26]. All experiments were repeated three independent times.

For the SRB assay [26], following treatment with *A. vulgaris* extract and PCT (as a positive control) for 72 h, cells underwent PBS washing before fixation with 10% TCA for 2 h at 4 °C. Subsequently, cells were rinsed in distilled water and stained with a 0.4% SRB solution for 30 min at RT. After the incubation period, the dye was dissolved in 1% acetic acid, washed, and left to dry overnight. Finally, the dye was then dissolved in 10 mM tris buffer for 20 min. The absorbance was measured using an automatic reader for microtiter plates (LKB 5060-006, LKB, Vienna, Austria) at 540/670 nm. The cell viability was expressed as a percentage of control, non-treated cells, which was arbitrarily set to 100%. All experiments were repeated three independent times. Dose–response curves were constructed through non-linear regression analysis in GraphPad Prism 10.1.2 software (San Diego, CA, USA). IC_50_ values, defined as 50% reduction of cell viability, were calculated using SigmaPlot 15 software and Microsoft Excel 2013 with the four-parametric logistic function.

Combined treatment with *A. vulgaris* extract and chloroquine (autophagy inhibitor) was carried out to evaluate the nature of the detected autophagy. The 4T1 cells were seeded overnight and treated with an IC_50_ concentration of *A. vulgaris* extract (45 μg/mL) and chloroquine (20 µM) simultaneously. After 72 h incubation, cell viability was evaluated using the SRB assay.

### 4.4. Detection of Apoptosis (Ann/PI Staining)

In order to detect apoptosis, 4T1 cells were seeded overnight and treated with an IC_50_ concentration of *A. vulgaris* extract (45 μg/mL). After 72 h incubation, the cells were trypsinized and stained with annexin V-FITC/PI (15 μg/mL) for 15 min at RT in the dark. Afterward, annV-binding buffer (ABB) was used for resuspension of the 4T1 cells. Finally, the cells were analyzed using flow cytometry (CyFlow^®^ Space Partec and PartecFloMax^®^ 2.52 software (Munster, Germany)) [69].

### 4.5. Activation of Caspases

For the detection of caspase activation, 4T1 cells were seeded overnight and exposed to an IC_50_ concentration of *A. vulgaris* extract (45 μg/mL). After the incubation period (72 h), the trypsinized cells were stained with pan-caspase inhibitor (apostat) and incubated for 30 min at 37 °C. Afterward, cells were then washed, resuspended in PBS, and analyzed with the CyFlow^®^ Space Partec flow cytometer using PartecFloMax^®^ 2.52 software (Munster, Germany) [69].

Alternately, 4T1 cells were seeded in a four-chamber slide (Millicell^®^ EZ SLIDES, Merck Millipore, Burlington, MA, USA) at 5 × 10^3^ cells/well density and left to adhere overnight, followed by exposure to *A. vulgaris* extract (IC_50_ concentration) and 72 h incubation. Finally, cells were stained via apostat staining and analyzed using fluorescence microscopy at 400× magnification with a Leica DM4 B with DFC7000 T microscope camera (Leica Microsystems CMS GmbH, Wetzlar, Germany).

### 4.6. Detection of Autophagy

The 4T1 cells were seeded overnight and exposed to an IC_50_ concentration of *A. vulgaris* extract (45 μg/mL). After the 72 h incubation period, the cells were stained for 15 min at 37 °C with 10 µM of AO solution. The dye was removed by washing with PBS. Finally, cells were resuspended using PBS and analyzed with the CyFlow^®^ Space Partec flow cytometer using PartecFloMax^®^ 2.52 software (Munster, Germany) [69].

Additionally, 4T1 cells were analyzed via fluorescence microscopy after treatment with an IC_50_ concentration of *A. vulgaris* extract. Cells were seeded overnight in the four-chamber slide (Millicell^®^ EZ SLIDES, Merck Millipore, Burlington, MA, USA) at 5 × 10^3^ cells/well density and exposed to *A. vulgaris* extract for 72 h. After the incubation period, the treatment was discarded and the cells were washed with PBS followed by a 15 min incubation with AO staining at a final concentration of 10 µM. Finally, the cells were washed with PBS and evaluated with fluorescence microscopy at 400× magnification using the Leica DM4 B with DFC7000 T microscope camera (Leica Microsystems CMS GmbH, Wetzlar, Germany).

### 4.7. Detection of Cell Proliferation

The 4T1 cells were prestained with CFSE (1 μM) and kept in the incubator for 10 min at 37 °C. After the incubation period, 10% FBS-RPMI medium was added and the cells were centrifuged, washed with PBS, resuspended in medium, seeded, and exposed to an IC_50_ concentration of *A. vulgaris* extract (45 μg/mL). After the 72 h incubation period, the 4T1 cells were washed with PBS, trypsinized, and analyzed with the CyFlow^®^ Space Partec flow cytometer using PartecFloMax^®^ 2.52 software (Munster, Germany) [69].

### 4.8. Assessment of Reactive Oxygen and Nitrogen Species (ROS/RNS) Production

Using redox-sensitive dye DHR123, production of ROS/RNS was detected. Prior to seeding, 4T1 cells were stained with DHR123 (1 μM) and incubated for 20 min at 37 °C. Afterward, cells were then exposed to an IC_50_ concentration of *A. vulgaris* extract. Following the 72 h incubation period, the cells were washed with PBS, trypsinized, and analyzed with the CyFlow^®^ Space Partec flow cytometer using PartecFloMax^®^ 2.52 software (Munster, Germany) [26].

In order to detect via fluorescence microscopy the production of ROR/RNS in 4T1 cells after exposure to *A. vulgaris* extract (IC_50_ concentration), cells were prestained with DHR123 to a final concentration of 1 µM, seeded in a four-chamber slide (Millicell^®^ EZ SLIDES, Merck Millipore, Burlington, MA, USA) at 5 × 10^3^ cells/well density, left to adhere overnight, and treated with *A. vulgaris* extract for 72 h. Finally, cells were washed with PBS and analyzed at 400× magnification using a Leica DM4 B with DFC7000 T microscope camera (Leica Microsystems CMS GmbH, Wetzlar, Germany).

### 4.9. Staining with Propidium Iodide (PI) on Chamber Slides

For fluorescence microscopy analysis, 4T1 cells were seeded overnight on sterile 4-chamber glass microscope slides (Millicell^®^ EZ SLIDES, Merck Millipore, Burlington, MA, USA) at a density of 3 × 10^4^ cells/well in 400 µL medium. Afterward, the 4T1 cells were then exposed to an IC_50_ concentration of *A. vulgaris* extract and incubated for 72 h. After incubation, the supernatant was discarded from the chambers, the cells were then washed with PBS, and they were then fixed in 4% PFA for 15 min at RT. Thereafter, the cells were washed in PBS several times, and stained with a solution of 50 μg/mL PI, 0.1 mM EDTA pH 8.0, 85 μg/mL RNase, and 0.1% Triton X-100 in PBS for 1–2 min. Finally, fluorescent mounting medium (Fluoromount-G) was used for coating the slides. A Zeiss AxioObserver Z1 inverted fluorescence microscope (Carl Zeiss AG, Oberkochen, Germany) was used for the analysis of the formed slides at 400 × magnification [70].

### 4.10. Clonogenic Assay—Colony Forming Assay

The assessment of the ability of 4T1 cells to form colonies in vitro after treatment with a subtoxic concentration of *A. vulgaris* extract involved evaluation using a clonogenic (colony formation) assay. The 4T1 cells were grown in a monolayer in 75 cm^2^ flasks, exposed to a subtoxic concentration (22.5 μg/mL) of *A. vulgaris* extract, and incubated for 72 h. Following the incubation period, the cells were trypsinized, counted, and seeded at a low number of cells per well (1 × 10^3^ cells/well) in 6-well plates. The 4T1 cells were allowed to grow and form colonies for 7 days, with the medium exchange on the fourth day. After the incubation period, the medium was discarded, and the formed colonies were washed using PBS and then fixed (4% PFA for 30 min at RT). Colonies were finally stained with 0.02% CV solution at RT for 15 min. Afterwards, cells were washed twice with PBS to remove the remaining dye. Colony quantification was performed using ImageJ analysis 1.51j8 software. In order to calculate the planting efficiency (PE) and the surviving fraction (SF), the mean value of the number of colonies obtained from three wells was used. PE, which is a measure of the number of colonies formed from individual cells, was calculated as the number of colonies counted divided by the number of planted cells × 100. SF was calculated as PE of treated cells divided by the PE of control cells × 100 and graphically represented as a percentage [69].

### 4.11. Wound Healing (Scratch) Assay

The scratch test, in which cells exhibit bidirectional migration from the edges of a scratch wound, was used for assessment of the cells’ motility. After reaching a confluence of 4T1 cells in the 6-well plates, a scratch was drawn with a sterile pipette tip (200 μL) across the center of the wells to make a clear wound area. After creating the wound, cells were washed using PBS several times in order to remove damaged cells and then exposed to a subtoxic dose (22.5 μg/mL) of *A. vulgaris* extract for 48 h. Following the incubation period, the cells were washed, fixed with 4% PFA for 30 min at RT, stained with 0.02% of CV solution for 15 min, and digitally photographed with a Nikon stereomicroscope (SMZ800N, Nikon Instruments Inc., Melville, NY, USA) at 6× magnification [69].

### 4.12. Assay for Quantification of Cell Adhesion In Vitro

For the assessment of cell adhesion in vitro, 4T1 cells were seeded in 75 cm^2^ flasks and treated with a subtoxic dose (22.5 μg/mL) of *A. vulgaris* extract. At 72 h, cells were trypsinized, harvested, and left for 30 min at RT to reconstruct cell membranes. Sterile 96-well plates were pre-coated with 20 μg/mL of Matrigel^®^ (50 μL/well) and left at 4 °C overnight to polymerize. Gel-coated wells were washed 3 times with PBS, and then the cells were seeded (3 × 10^4^ cells/well). In parallel, cell adhesion was estimated on plastic (96-well plates not coated with Matrigel^®^). Thereafter, the cells were kept at 37 °C for 1 h to adhere. Cells that did not attach in this time frame were removed by PBS washing, while the number of remaining adherent cells was determined by SRB assay. Relative cell adhesion was assessed by measuring the absorbance at 570/670 nm using an automatic microtiter plate reader [69].

### 4.13. Assays for Quantification of Cell Migration and Invasion In Vitro

For quantitative assessment of the influence of *A. vulgaris* extract treatment on the migration and invasive potential of 4T1 cells in vitro, transmigration and invasive assays were used. For these purposes, inserts with a diameter of 6.4 mm and a pore diameter of 8 μm were placed in sterile 24-well plates. In order to conduct the invasion assay, the upper membrane surface of these inserts was covered with a thin layer of Matrigel^®^ (500 μg/mL in PBS, 20 μL/membrane) that was allowed to polymerize at 4 °C overnight, while for the migration assay, the membrane remained uncovered with Matrigel^®^. The 4T1 cells were grown in 75 cm^2^ flasks and exposed to a subtoxic dose of *A. vulgaris* extract (22.5 μg/mL) for 72 h. Before the 4T1 cells were seeded, the polymerized gel-coated inserts were washed twice with PBS. After incubation, the cells were trypsinized, then 7 × 10^4^ cells were resuspended in 200 μL of 0.1% BSA-RPMI medium and seeded in the upper part of each well. As a chemoattractant for the cells, 10% FBS-RPMI was used, which was poured from the outside of the chambers, precisely into the wells of the cell culture plate. The 24-well plates were further incubated at 37 °C for 3 h to assess invasiveness and migration. Cells that did not migrate through the pores of the membrane (in the migration assay) or the extracellular matrix layer (in the invasion assay) were meticulously eliminated using cotton swabs. Conversely, invading or migrating cells underwent fixation with 4% PFA for 10 min and were then stained with a 0.02% CV solution for 20 min at RT. Following staining, the cells were washed with H_2_O, air-dried, and subsequently, the membranes were detached from the chambers and mounted on microscope slides. Cells were observed and digitally photographed using the ZOE fluorescent cell imager (Bio-Rad Laboratories, Hercules, CA, USA). The average cell count from 5 randomly chosen independent fields on each membrane is shown. Each experiment was conducted in triplicate [69].

### 4.14. Induction of 4T1 Cells and Experimental Treatment In Vivo

In order to evaluate the effect of *A. vulgaris* extract in vivo, tumors were induced by orthotopic implantation of 4T1 cells (2 × 10^4^ in 50 μL PBS) into the fourth right mammary fat pad of female mice of the BALB/C strain. Upon tumor appearance (10 days after tumor induction) the mice were treated with *A. vulgaris* extract by i.p. injection (50 mg/kg body weight in 4% DMSO/PBS), with a regimen of 5 days treatment, 2 days break. The control group underwent treatment with a vehicle (4% DMSO/PBS). The animals were sacrificed 23 days after tumor implantation, the tumors were collected, and their volume was calculated using the formula: (length × width^2^) × 0.52.

### 4.15. Histopathology

After 23 days of in vivo experiment, animals were sacrificed via cervical dislocation and tumors, kidneys, and livers were macroscopically examined, isolated, and fixed in 10% buffered formalin of neutral pH value, for 24 h. All tissues were afterwards sectioned through the largest tissue plane and additionally fixed for 24 h. Tissue processing was carried out in an automated tissue processor (Milestone SRL LOGOS ONE, Sorisole, BG, Italy). Afterwards, using the embedding console (SAKURA Tissue-Tek TEC 5, Sakura Finetek, Torrance, CA, USA), tissues were embedded in paraffin blocks. The thickness of tissue slices was 4 µm and was obtained with a microtome LEICA RM 2245 (Leica Biosystems, Nussloch, Germany). Then, the tissue slices were mounted on glass slides and hematoxylin–eosin (H/E) staining was applied using the MYREVA SS-30H automated slide stainer (Especialidades Médicas MYR, S.L., Tarragona, Spain). For histopathological analysis of slides, an Olympus BX43 microscope (OLYMPUS EUROPA HOLDING GMBH, Hamburg, Germany) was used. For analysis and documentation purposes, a Leica Aperio AT2 slide scanner (Leica Biosystems, Nussloch GmbH, Germany) was additionally used. Furthermore, for morphometrical analysis of virtual slides, Leica AperioImageScope (version 12.4.6, Leica Biosystems, Nussloch GmbH, Germany) and FIJI-ImageJ 1.51j8 software were used [69].

### 4.16. Western Blot Analysis

The 4T1 cells were treated with the IC_50_ concentration of *A. vulgaris* extract for 6, 12, 18, 24, 48, and 72 h periods. After incubation, the cells were lysed with a protein lysis buffer which contained 62.5 mM Tris–HCL pH 6.8, 10% glycerol, 50 mM dithiothreitol, and 2% *w*/*v* SDS. Lowry’s method was used for measuring protein content. Afterwards, cell lysates (30 μg) were separated on a 12% SDS–polyacrylamide gel with a protein molecular weight marker (prestained PageRuler ladder (Thermo Fisher Scientific, Waltham, MA, USA)). Proteins were then transferred to a polyvinylidene difluoride (PVDF) membrane using a semi-dry transfer (Fastblot B43; BioRad, Göttingen, Germany). After transfer, the membranes underwent blocking with 5% BSA in PBS containing 0.1% Tween 20 for 1 h at RT and were then probed with a primary antibody overnight at 4 °C. Following washing, the membranes were incubated with secondary antibodies (goat anti-rabbit IgG-HRP, and bovine anti-mouse IgG-HRP (Santa Cruz Biotechnology, Dallas, TX, USA)) for 1 h [69]. The presence of bands was confirmed with an iBright™ FL1500 Imaging System (Thermo Fisher Scientific, Waltham, MA, USA) and analyzed using iBright Analysis 5.2.1 Software (Thermo Fisher Scientific, Waltham, MA, USA). In this study, the following primary antibodies were used: β-actin (Abcam, Cambridge, UK), integrin β-1, FAK, vinculin, and α-smooth muscle actin (α-SMA) (Elabscience Biotechnology Inc., Huston, TX, USA).

### 4.17. Preparation of Single-Cell Suspensions and Flow Cytometric Analysis

In order to obtain the single-cell suspensions of the spleen, mechanical dispersion was used. At the same time, as previously described [71], enzymatic digestion was used in order to obtain the single-cell suspensions of primary tumors. Anti-mouse antibodies labeled with fluorochrome and specific for CD11c, CD40, MHCII, CD86, CD3, CD8, PD-1, or isotype-matched controls (BD Pharmingen, NJ/Invitrogen, Carlsbad, CA, USA) were used. For intracellular staining, phorbol 12-myristate 13-acetate (50 ng/mL, Sigma-Aldrich, St. Louis, MO, USA), ionomycin (500 ng/mL, Sigma-Aldrich), and GolgyStop (BD Pharmingen, Franklin Lakes, NJ, USA) were used for stimulation for 4 h. Finally, cells were stained with anti-mouse antibodies labeled with fluorochrome specific for perforin, CD107a, and granzyme (BD Pharmingen/ BioLegend/eBiosciences), and 2 × 10^4^ to 5 × 10^4^ cells were acquired for FACS analysis. Flow cytometry was carried out using a FACSCalibur flow cytometer (BD Biosciences, San Jose, CA, USA), and the data were analyzed with FlowJo 10.0.7r2 Software.

### 4.18. Statistical Analysis

The statistical package Statistica 12 (Informer Technologies, Inc., Los Angeles, CA, USA) was used for data analysis. The significance between groups was evaluated by Student *t*-test or alternatively by one-way ANOVA followed by Tukey’s honest significant difference (HSD) post hoc test, and two-sided *p* values of less than 0.05 were considered to indicate statistical significance. For in vivo experiments, a non-parametric Mann–Whitney U test was used.

## 5. Conclusions

The antitumor potential of the ethanolic extract of *A. vulgaris* assessed in the TNBC model in vitro and in vivo presents a panel of well-orchestrated activities influencing neoplastic cell viability, metastatic potential, and expression of proteins involved in ECM and intracellular cytoskeleton crosstalk. The overall efficacy of this herbal extract is further connected with its ability to provoke immunogenicity of tumor cells through increased dendritic cell antigen-presenting potential and the quantity/efficacy of cytotoxic lymphocytes in the tumor microenvironment and spleen, thus limiting tumor progression and dissemination. Accordingly, the *A. vulgaris* extract efficiently suppressed tumor growth in syngeneic BALB/C mice through multiple activities at local and systemic level, relevant for tumor progression and spreading, suggesting the importance of further investigation in the field of advanced cancer biology and treatment. Regardless of the fact that this and other extracts cannot be accepted as standard therapy due to their complexity as well as the lack of strict quality control during preparation, the results of this study can be a good basis for the identification of new components or combined treatments whose effect can be amplified by new delivery strategies such as nanotechnology. The obvious immunogenic effect of the applied treatment can serve as a sensitizer for standard therapeutics through combined protocols or neoadjuvant therapies. 

## Figures and Tables

**Table 1:** Data represent the IC_50_ values calculated after 72 h treatment with *A. vulgaris* ethanolic extract and PCT, and are expressed as mean ± SD.

	Assays	4T1	MDA-MB-468	MDA-MB-361	MDA-MB-231
*A. vulgaris* extract (µg/mL)	MTT	41.8 ± 3.3	65.6 ± 1.3	71.2 ± 1.9	122.2 ± 4.7
	SRB	49.3 ± 3.0	71.9 ± 3.8	61.8 ± 2.3	160.4 ± 6.0
PCT (ng/mL)	MTT	12.0 ± 1.0	2.0 ± 0.6	3.1 ± 0.2	8.4 ± 0.7
	SRB	31.0 ± 0.6	2.7 ± 0.1	3.9 ± 0.3	9.4 ± 0.9

## Data Availability

Data supporting the obtained results can be obtained from the authors upon request.

## Supplementary Materials

The following supporting information can be downloaded at: https://www.mdpi.com/article/10.3390/ph17030286/s1, Figure S1: *A. vulgaris* extract decreased the viability of all cell lines in vitro; Figure S2: *A. vulgaris* extract provoked moderate caspase activation in the 4T1 cell line; Figure S3: *A. vulgaris* extract induced the production of ROS/RNS in the 4T1 cell line; Figure S4: Representative FACS plots and histograms; Table S1: Mouse urine parameters.
