# Supplementary material for: Dual Role of Alchemilla vulgaris L. Extract in Breast Cancer Regression: Reestablishment of Effective Immune Response
Source: Pharmaceuticals (Basel). 2024 Feb 23;17(3):286. doi: 10.3390/ph17030286 (PMC10975144; doi:10.3390/ph17030286)
Supplement: Supplementary file 1 [file pharmaceuticals-17-00286-s001.zip › pharmaceuticals-2799472-supplementary.pdf]

# SUPPORTING INFORMATION:

*Article*

# Dual Role of *Alchemilla vulgaris* L. Extract in Breast Cancer Regression: Reestablishment of Effective Immune Response

**Sanja Jelača [1], Ivan Jovanovic [2], Dijana Bovan [1], Marina Z. Jovanovic [3], Milena M. Jurisevic [4], Duško Dunđerović [5], Zora Dajic-Stevanovic [6], Nebojsa Arsenijevic [2], Sanja Mijatović [1,*] and Danijela Maksimović-Ivanić [1,*]**

1 Department of Immunology, Institute for Biological Research "Siniša Stanković"—National Institute of the Republic of Serbia, University of Belgrade, Bulevar Despota Stefana 142, 11108 Belgrade, Serbia; sanja.jelaca@ibiss.bg.ac.rs (S.J.); dijana.draca@ibiss.bg.ac.rs (D.B.)
2 Center for Molecular Medicine and Stem Cell Research, Faculty of Medical Sciences, University of Kragujevac, Svetozara Markovića 69, 34000 Kragujevac, Serbia; ivanjovanovic77@gmail.com (I.J.); nebojsa_arsenijevic@yahoo.com (N.A.)
3 Department of Otorinolaringology, Faculty of Medical Sciences, University of Kragujevac, 34000 Kragujevac, Serbia; marina_jovanovic@rocketmail.com
4 Department of Pharmacy, Faculty of Medical Sciences, University of Kragujevac, 34000 Kragujevac, Serbia; milena.jurisevic13@gmail.com
5 Institute of Pathology, School of Medicine, University of Belgrade, Dr Subotića 8, 11000 Belgrade, Serbia; dusko.dundjerovic@med.bg.ac.rs
6 Faculty of Agriculture, University of Belgrade, Nemanjina 6, 11080 Belgrade, Serbia; dajic@agrif.bg.ac.rs
* Correspondence: sanjamama@ibiss.bg.ac.rs (S.M.); nelamax@ibiss.bg.ac.rs (D.M.-I.); Tel.: +381-11-2078452 (S.M. & D.M.-I.)

**List of content:**

**Table S1**: Mouse urine parameters.
**Figure S1.** *A. vulgaris* extract decreased the viability of all cell lines in vitro.
**Figure S2.** *A. vulgaris* extract provoked moderate caspase activation in 4T1 cell line.
**Figure S3.** *A. vulgaris* extract induced the production of ROS/RNS in 4T1 cell line.
**Figure S4.** Representative FACS plots and histograms.

**Table S1**. Mouse urine parameters (mean ± SD).

| | Control | *A. vulgaris* extract |
|---|---|---|
| Spec. gravity | 1030 ± 0 | 1028.9 ± 2.2 |
| pH | 5.3 ± 0.5 | 5.6 ± 0.5 |
| Glucose (mg/100mL) | negative | negative |
| Nitrite | negative | negative |
| Protein (mg/100mL) | 14.4 ± 8.8 | 15 ± 9.2 |
| Ketones (mg/100mL) | 6.4 ± 2.4 | 5 ± 0 |
| Urobilinogen (mg/100mL) | 2.1 ± 1.5 | 1 ± 0 |
| Bilirubin (mg/100mL) | negative | negative |
| Blood (RBC/μL) | negative | negative |
| Leukocytes (WBC/μL) | negative | negative |

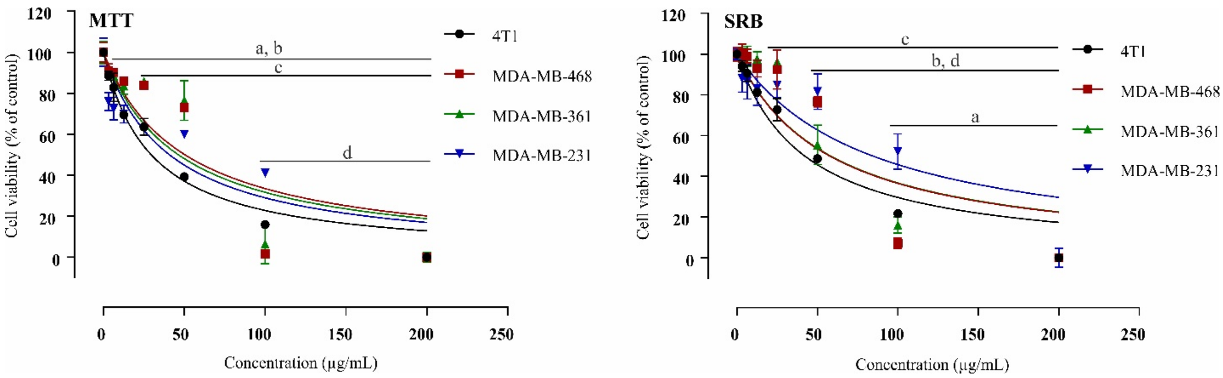


**Figure S1.** *A. vulgaris* extract decreased the viability of all cell lines in vitro. Cells were exposed to a wide range of doses of *A. vulgaris* extract for 72 h. After incubation, MTT and SRB assays were performed. All data are presented as mean ± SD from one representative out of three independent experiments and statistically significant were considered *p* values less than 0.05, comparing to controls (a – MDA-MB-231; b – MDA-MB-468; c – 4T1; d – MDA-MB-361).

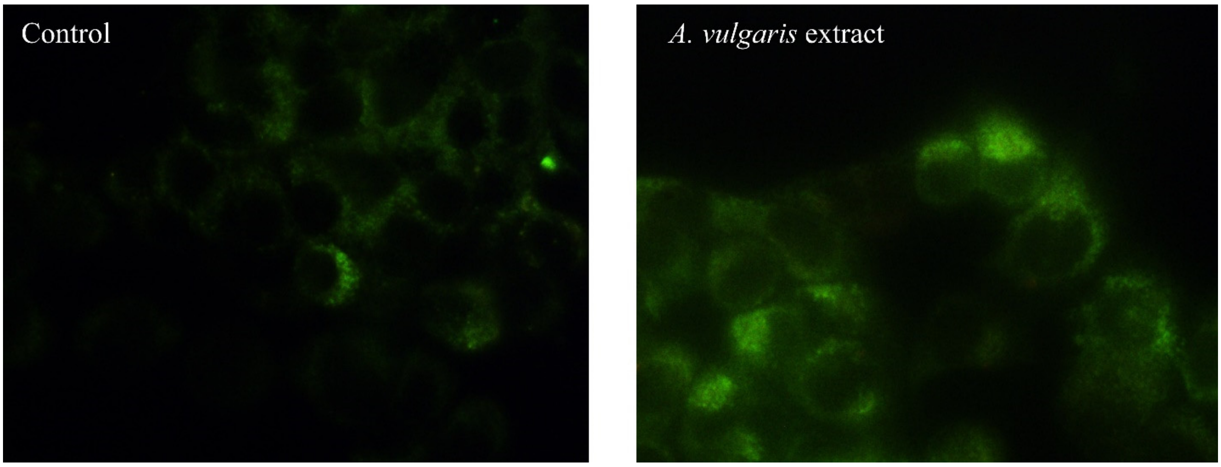


**Figure S2.** *A. vulgaris* extract provoked moderate caspase activation in 4T1 cell line. Cells exposed to an $IC_{50}$ concentration of *A. vulgaris* extract for 72 h followed by apostat staining and fluorescence microscopy at 400× magnification.

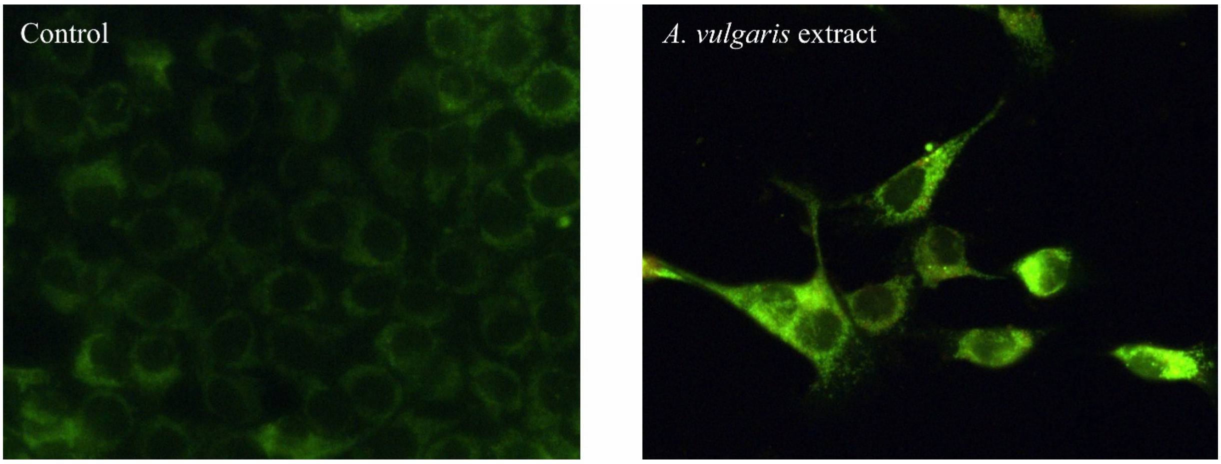


**Figure S3.** *A. vulgaris* extract induced the production of ROS/RNS in 4T1 cell line. Cells were prestained with DHR123 and exposed to an $IC_{50}$ concentration of *A. vulgaris* extract for 72 h. After the incubation period, fluorescence microscopy was performed with 400× magnification.

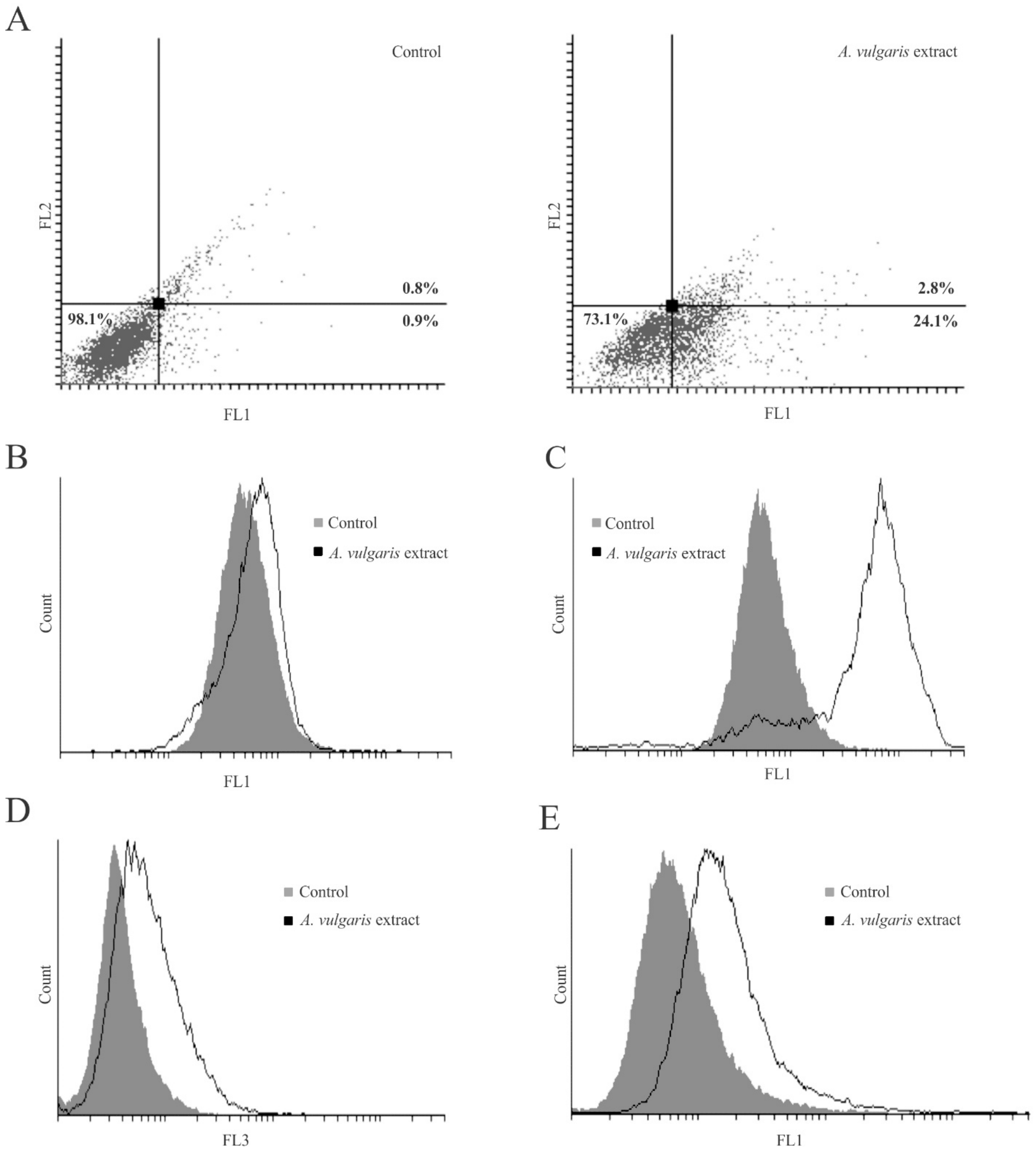


**Figure S4.** Representative FACS plots and histograms. Ann/PI staining (A); Apostat staining (B); DHR123 staining (C); Acridin orange (AO) staining (D); CFSE staining (E).
